# Supplementary material for: Demonstrating the potential of yttrium-doped barium zirconate electrolyte for high-performance fuel cells
Source: Nat Commun. 2017 Feb 23;8:14553. doi: 10.1038/ncomms14553 (PMC5331335; doi:10.1038/ncomms14553)
Supplement: Supplementary Information — Supplementary Figures, Supplementary Table, Supplementary Notes, Supplementary Methods and Supplementary References. [file ncomms14553-s1.pdf]

Supplementary Table 1. Comparison with PCFCs based on acceptor doped barium zirconate electrolytes in the literature.

Type	Electrolyte	Electrolyte thickness [μm]	Fabrication technique	Anode/ Cathode	OCV [V]	PPD [mW cm^{-2}]	ASR_{ohm} [Ω cm^2]	ASR_{pol} at OCV [Ω cm^2]	Temp. [°C]	Ref.
Free-standing	$BaZr_{0.8}Y_{0.2}O_{3-\delta}$	0.13	PLD	Pt/Pt	1.12	120	-	-	450	1
	$BaZr_{0.8}Y_{0.2}O_{3-\delta}$	0.11	ALD	Pt/Pt	1.09	136	-	-	400	1
	$BaZr_{0.8}Y_{0.2}O_{3-\delta}$ (3D crater)	0.12	PLD	Pt/Pt	0.85	186	-	-	450	2
	$BaZr_{0.8}Y_{0.2}O_{3-\delta}$ (cup-shape)	0.30	PLD	Pt/Pt	0.56	7.6	-	-	400	3
	$BaZr_{0.85}Y_{0.15}O_{3-\delta}$	0.2	PLD	Pt/Pt	1.08	27.2	-	-	475	4
AAO-supported	$BaZr_{0.8}Y_{0.2}O_{3-\delta}$	1	PLD	Pt-Pd/Pt	1	9.1	0.42	622	400	5
	$BaZr_{0.8}Y_{0.2}O_{3-\delta}$	1.34	PLD	Pt/Pt	1.1	21	0.22	-	450	6
Anode-supported	$BaZr_{0.8}Y_{0.2}O_{3-\delta}$	4	PLD	Ni-BZY/ LSCF-BCYb	0.99	110	1.85	0.56	600	7
	$BaZr_{0.8}Y_{0.2}O_{3-\delta}$	25	Dip-coating	Ni-BCZY/ SSC-SDC	0.97	55	3.24	1.98	600	8
	$BaZr_{0.8}Y_{0.2}O_{3-\delta}$	20	Co-pressing	Ni-BCZY/ SSC-SDC	1.014	70	1.4	1.3	600	9
	$BaZr_{0.8}Y_{0.2}O_{3-\delta}$	30	Co-pressing	Ni-BZY/ LSCF-BZPY	0.98	51	1.12	3.18	600	10
	$BaZr_{0.8}Y_{0.2}O_{3-\delta}$	30	Co-pressing	Ni-BZY/ BSCF	0.94	45	-	-	700	11
	$BaZr_{0.9}Y_{0.1}O_{3-\delta}$	20	Co-pressing with ionic diffusion	Ni-BZY/ PBC-BZPY	0.99	169	0.88	0.26	600	12
	$BaZr_{0.8}Y_{0.2}O_{3-\delta}$	5	Aerosol deposition	Ni-YSZ/ LSCF-GDC	0.96	83	0.92	1.19	600	13
	$BaZr_{0.8}Y_{0.2}O_{3-\delta}$	~16	Co-pressing	Ni-BCZY/ SSC-SDC	1.00	61	-	-	600	14
	$BaZr_{0.8}Y_{0.2}O_{3-\delta}$ -Li	25	Co-pressing	Ni-BZY/ BSCF-BZY	0.98	53	0.95	1.66	700	15
	$BaZr_{0.7}Pr_{0.1}Y_{0.2}O_{3-\delta}$	20	Co-pressing	Ni-BZY/ LSCF-BZPY	0.96	81	1.33	1.3	600	16

$BaZr_{0.8}Y_{0.2}O_{3-\delta}$ -CaO	25	Co-pressing	Ni-BZY/ LSCF-BZY	0.99	141	0.95	1.66	700	[17]
$BaZr_{0.7}Pr_{0.1}Y_{0.2}O_{3-\delta}$	12	Co-pressing	Ni-BZY/ LSCF-BZP3Y	0.97	163	0.53	0.53	600	[18]
$BaZr_{0.8}In_{0.2}O_{3-\delta}$	20	Co-pressing	Ni-BCZY/ SSC-SDC	1.01	34	3.53	4	600	[19]
$BaZr_{0.7}In_{0.3}O_{3-\delta}$	15	Co-pressing	Ni-BZI/ PBC-BZPY	0.946	84	2.01	0.27	600	[20]
$BaZr_{0.7}Nd_{0.1}Y_{0.2}O_{3-\delta}$	30	Co-pressing	Ni-BZNY/ BSCF	0.99	105	1.46	-	600	[21]
$BaZr_{0.84}Y_{0.15}Cu_{0.01}O_{3-\delta}$	10	LCP	Ni-BZYCu/ LSCF-BZY	0.985	28.2	-	-	650	[22]
$BaZr_{0.7}Sn_{0.1}Y_{0.2}O_{3-\delta}$	12	Drop coating	Ni-BZSY/ SSC-SDC	0.98	214	0.46	0.41	600	[23]
$BaZr_{0.8}Y_{0.15}In_{0.05}O_{3-\delta}$	12	Drop coating	Ni-BZY/ SSC-SDC	0.99	221	0.58	0.5	600	[24]
$BaZr_{0.8}Y_{0.16}Zn_{0.04}O_{3-\delta}$	20	Spin coating	Ni-BZY/ Pt	0.94	75	1.15	2.6	600	[25]
$BaZr_{0.85}Y_{0.15}O_{3-\delta}$	2.5	PLD	Ni-BZY/ LSC	1.02	740	0.09	0.16	600	This work

OCV: open-circuit voltage, PPD: peak power density, ASR: area-specific resistance, PLD: pulsed laser deposition, ALD: atomic layer depo sition, LCP: liquid condensation process, AAO: anodic aluminum oxide

LSCF: $La_{0.6}Sr_{0.4}Co_{0.2}Fe_{0.8}O_{3-\delta}$, BCYb: $BzCe_{0.9}Yb_{0.1}O_{3-\delta}$, BCZY: $BaCe_{0.7}Zr_{0.1}Y_{0.2}O_{3-\delta}$, SSC: $Sm_{0.5}Sr_{0.5}CoO_{3-\delta}$, SDC: $Ce_{0.8}Sm_{0.2}O_{2-\delta}$, BZP3Y: $BaZr_{0.5}Pr_{0.3}Y_{0.2}O_{3-\delta}$, BSCF: $Ba_{0.5}Sr_{0.5}Co_{0.8}Fe_{0.2}O_{3-\delta}$, PBC: $PrBaCo_2O_{5+\delta}$, YSZ: $Zr_{0.84}Y_{0.16}O_{2-\delta}$, GDC: $Gd_{0.1}Ce_{0.9}O_{2-\delta}$, LSC: $La_{0.6}Sr_{0.4}CoO_{3-\delta}$

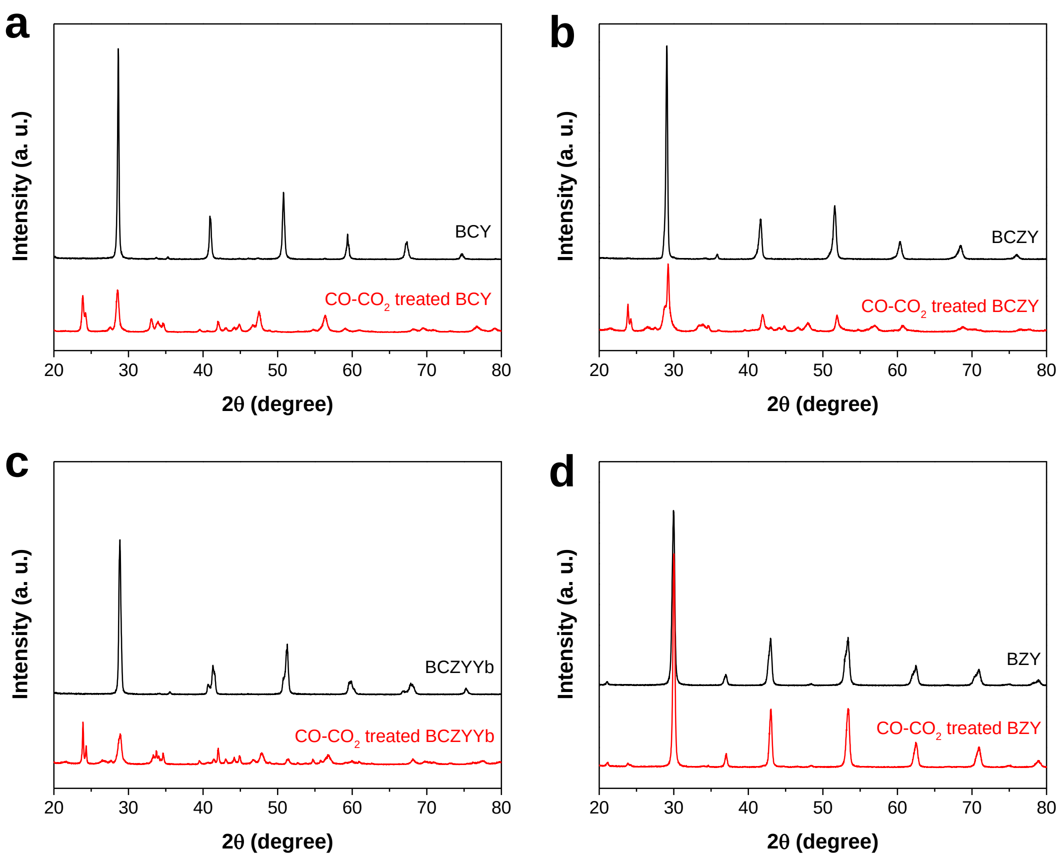

Supplementary Figure 1. Chemical stability of representative PC materials. X-ray diffraction patterns of: **a**, $BaCe_{0.8}Y_{0.2}O_{3-\delta}$ (BCY); **b**, $BaCe_{0.7}Zr_{0.1}Y_{0.1}Yb_{0.1}O_{3-\delta}$ (BCZYYb); **c**, $BaCe_{0.55}Zr_{0.3}Y_{0.15}O_{3-\delta}$ (BCZY); and **d**, $BaZr_{0.85}Y_{0.15}O_{3-\delta}$ (BZY) powders before and after exposure to 50% CO-50% CO_2 atmosphere at 600 °C for 150 h.

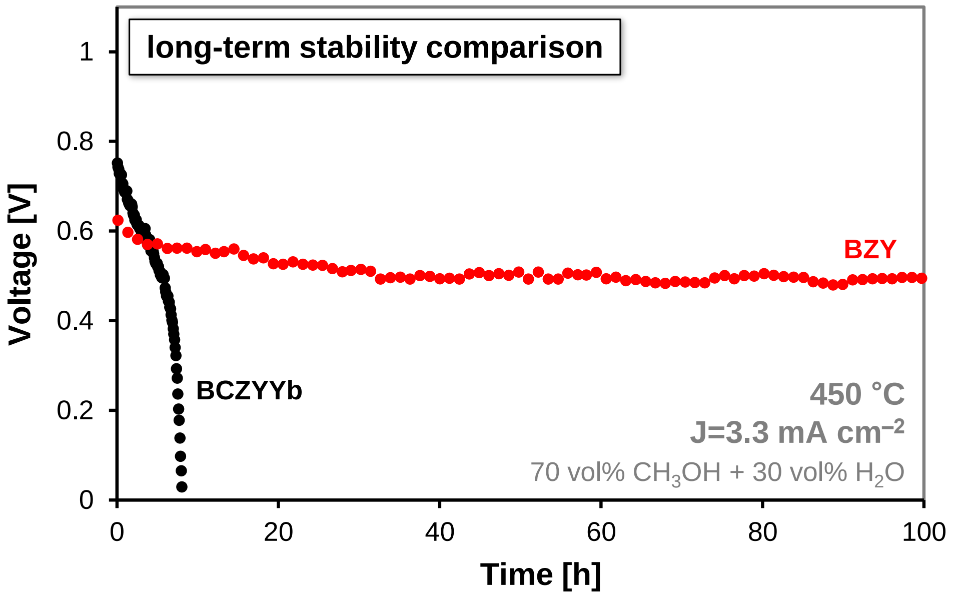

Supplementary Figure 2. Long-term stability with methanol fuel. Comparison of long-term stability of $BaZr_{0.85}Y_{0.15}O_{3-\delta}$ (BZY) and $BaCe_{0.7}Zr_{0.1}Y_{0.1}Yb_{0.1}O_{3-\delta}$ (BCZYYb) under fuel cell operating conditions of flowing vaporized methanol with water as a fuel. Note that the relatively low power outputs from these cells compared to ones from the anode-supported thin-film BZY cells of the main article is due to the relatively large thickness of the electrolyte (about 750 μm) and the use of methanol fuel.

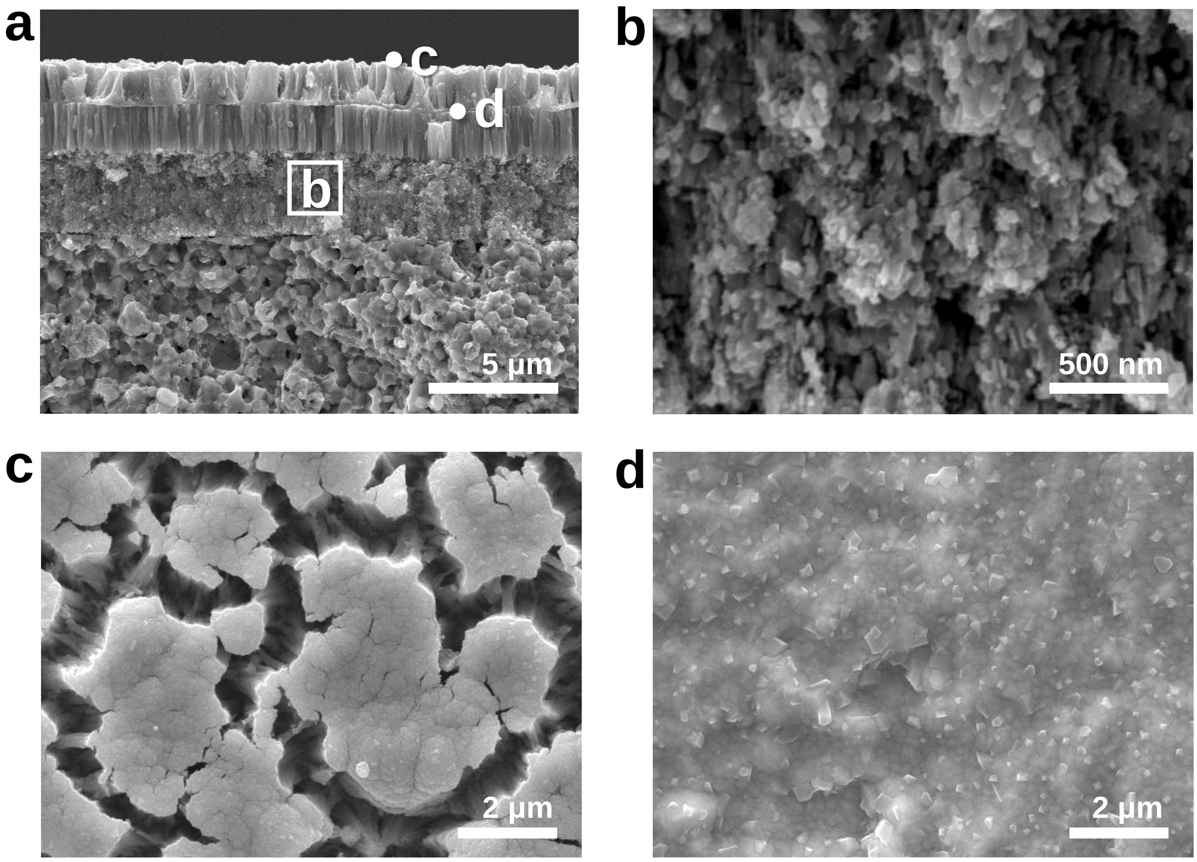

Supplementary Figure 3. SEM of the BZY PCFC after optimization. a, Cross-sectional SEM image of the PCFC fabricated under the optimal conditions of Ni–BZY nano-AFL (containing 50 vol% Ni contents and post-annealed at 1300 °C). Four layers are apparently shown in order of porous LSC cathode, dense BZY electrolyte, porous Ni–BZY nano- and micron-AFLs from the top layer. **b**, A high magnification SEM image of Ni–BZY nano-AFL at the marked area in (**a**). **c**,**d**, Surface SEM images of LSC cathode (**c**) and BZY electrolyte (**d**), respectively.

Supplementary Note 1. Microstructural optimization of Ni–BZY nano-AFL

In order to successfully lower the large ohmic resistance of BZY-PCFCs and achieve high performance, reducing thickness of BZY electrolytes on the porous anodes is necessary without decrease in density. It was found that key in accommodation of thin and dense BZY electrolytes is optimization of nano-AFL fabricated on the tape-cast anode substrates (Fig. 2 a,b) determining the surface quality of electrolyte deposition and integrity between the thin electrolytes and the anode supports. Among many conditions for nano-AFL fabrication, composition of Ni and BZY and post-annealing temperature are demonstrated as critical factors for physical stability and electrochemical performance in the cell configuration.

The morphology changes of nano-AFL are plotted as a function of the Ni content and annealing temperature in supplementary Fig. 4a–j. It is apparent that grain growth and consequent interconnection are accelerated by increase in the annealing temperature, as shown in the SEM images moving from the as-deposited (supplementary Fig. 4a) to post-annealed at 1300 °C nano-AFL surfaces (supplementary Fig. 4d,g,j). On the other hand, pore coarsening is also promoted with increase in annealing temperature. These coarsened pores are origins of microstructural defects in the dense thin electrolyte grown by PLD.[26] It is also found that increase of Ni contents in nano-AFLs reduces the pore coarsening, more obviously observed in the SEM images obtained from nano-AFLs at 1300 °C. Thus, it seems to be needed a sensitive optimization between the Ni content and the annealing temperature to balance these counteracting effects.

Another factor that should be considered to decide the optimal microstructure of composite anodes is the morphology change after reduction of NiO to Ni, and the reduced surfaces of nano-AFLs are presented in supplementary Fig. 5a–j, corresponding to the morphology changes in supplementary Fig. 4. Severe Ni coarsening and the formation of white snowball-like particles are observed from the surface of non-annealed nano-AFL with a

Ni content of 50 vol% (supplementary Fig. 5a). Such intense agglomeration after reduction of NiO was also observed in samples annealed at relatively low temperatures (1100 °C) regardless of the Ni content. Nano-AFLs with a high Ni content (60 vol%) need higher annealing temperatures than the targeted range because Ni coarsening, even though the size is much reduced, is still shown in the sample annealed at 1300 °C. The Ni coarsening, which is critical in microstructural failures (such as delamination of the electrolyte at the anode surface, which results in poor OCV or fuel cell power output), is to be avoided by sufficient grain growth and optimizing the Ni content[27, 28]. Hence, it is clear that annealing at sufficiently high temperatures (≥ 1200 °C) and limiting the Ni content in the Ni-BZY interlayer (≤ 50 vol%) are necessary to avoid Ni coarsening during anode reduction, and the condensed conditions inside the red box in supplementary Fig. 5 will be considered for the next discussion of electrochemical optimization.

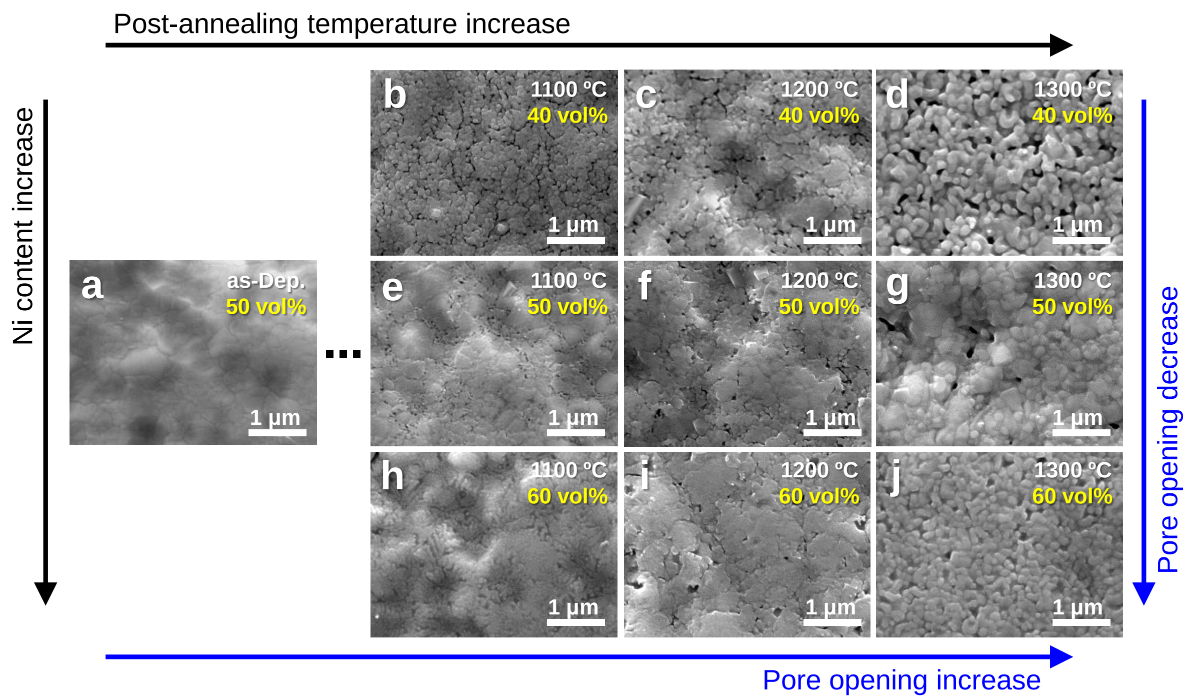

Supplementary Figure 4. Microstructural map of nano-AFLs. a–j, Surface SEM images of the NiO–BZY nano-AFLs fabricated under varying Ni contents of 40, 50, and 60 vol% and varying post-annealing temperatures of non-annealed (as-deposited), 1100, 1200, and 1300 °C.

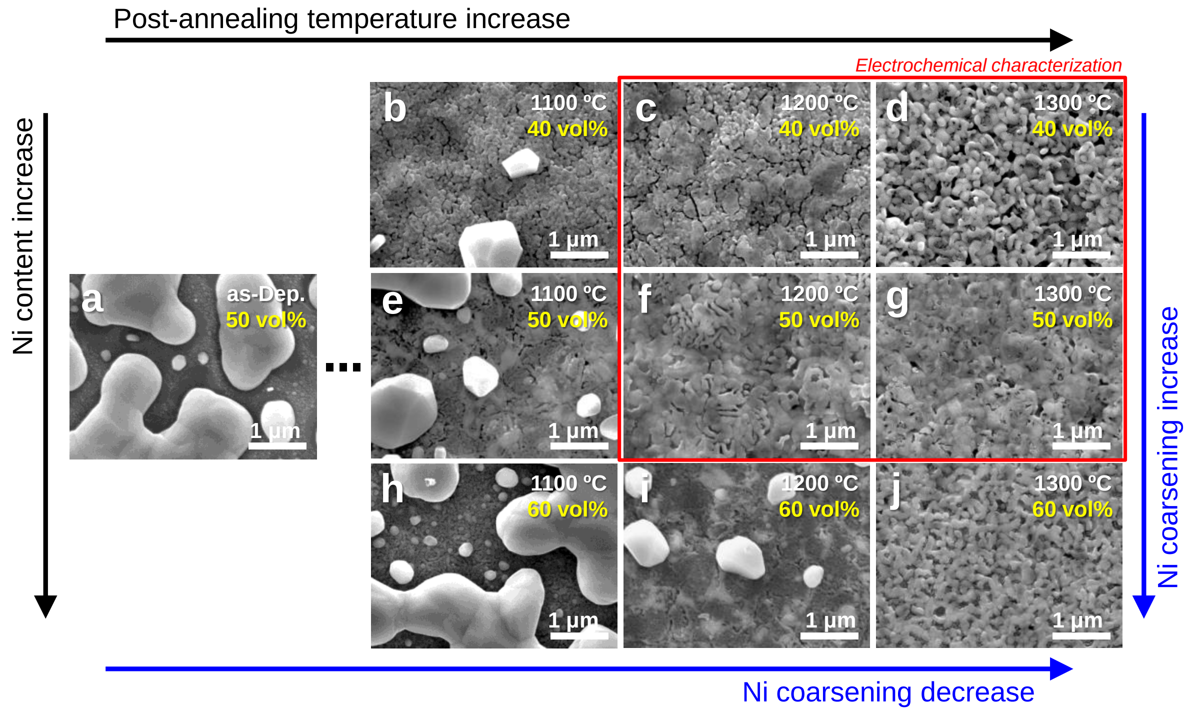

Supplementary Figure 5. Microstructural map of nano-AFLs after reduction. a–j, Surface SEM images of the NiO–BZY nano-AFLs after reduction in hydrogen corresponding the images in supplementary Fig. 4.

Supplementary Note 2. Electrochemical optimization of BZY-PCFCs with the optimal nano-AFLs

With the optimal Ni–BZY nano-AFLs decided by the microstructural optimization above, PCFCs were fabricated under the proposed fuel cell configuration in the main manuscript and their electrochemical performance was compared in this section. For convenience, the PCFC samples are identified with the Ni concentration and the annealing temperature used in the formation of nano-AFL. For example, 50-1200 PCFC represents a PCFC with nano-AFL containing 50 vol% Ni annealed at 1200 °C.

The OCV profiles from 50-1200 and 50-1300 PCFCs are discussed in Fig. 3a in the main manuscript as bad (non-optimized PCFC) and good (optimized PCFC) cases, respectively. The OCV profiles from 40-1200 and 40-1300 PCFCs are depicted in supplementary Fig. 6a. Apparently low OCVs compared with that of 50-1300 PCFC were obtained after the reduction step from the both PCFCs, but the irreversible OCV drop observed from 50-1200 PCFC is not shown. Reliability comparison of the OCV achievements of each PCFC setup is shown in supplementary Fig. 6b, extended from Fig. 3b as including the information of 40-1200 and 40-1300 PCFCs. As discussed in the main manuscript, 50-1200 PCFCs show low OCVs due to the poor physical integrity between the nano-AFL and the anode support. 40-1300 PCFCs also show badly scattered OCVs, as shown in supplementary Fig. 6b, which can be explained by formation of pore coarsening in nano-AFL after post-annealing. The relatively large pores developed on the surface of 40-1300 nano-AFL (supplementary Fig. 4d and 5d) is detrimental so that the electrolyte contains more defects after deposition and can easily break when larger pores are generated upon anode reduction, especially when a thin electrolyte is used, as is the case in this study. The 10 vol% increase of Ni content is helpful to reduce the pore size on the surface of post-annealed nano-AFL, as mentioned previously. Thus, high OCVs could be achieved from 50-1300 PCFCs

despite of annealing at the same temperature. Unlike the poorly reproducible OCVs of 40-1300 PCFCs, comparably high OCVs were obtained from 40-1200 PCFCs. It is because that the pore coarsening by post-annealing at 1200 °C is not as much as that at 1300 °C, providing deposition surface favorable to the thin BZY electrolyte fabrication. From this, we understand that the Ni content and annealing temperatures are to be carefully determined because a 10% change in the Ni content or 100 °C change in annealing temperature can cause a substantial impact on the structural integrity of the cell.

As results of the OCV comparison, we can reduce the optimizing conditions to 40-1200 and 50-1300 PCFCs, and the representative power curves obtained from each PCFC are compared in supplementary Fig. 7a. 50-1300 PCFC shows high power outputs with peak power density (PPD) of as much as 740 mW cm^2 at 600 °C, while much lowered power outputs were achieved in 40-1200 PCFC with PPD of 397 mW cm^2. The relatively low OCV initially leads to the lower performance of 40-1200 PCFC, but the rapid drop of power under about 0.6 voltage is also attributed to the decrease of PPD. The different cell resistances are clearly observed in AC impedance spectra in supplementary Fig. 7b. The impedance at low frequency is much large in 40-1200 PCFC compared with that in 50-1300 PCFC. Considering only the nano-AFL is differed between the two PCFCs, the different impedance should result from the anodic polarization resistance, and indeed, the differed impedance is in good agreement with the frequency range dominant by the mass transport and electrode charge transport in anode.[29, 30, 31]

Supplementary Fig. 7c,d show the focused ion beam (FIB) cross sectional morphologies focusing on the nano-AFLs of 40-1200 and 50-1300 PCFCs after the fuel cell test, respectively. Almost 5 times larger pores appeared in the interlayer of the 50-1300 PCFC than ones in the 40-1200 PCFC. This different pore sizes support our observation in the EIS comparison, which indicates that the impedance related to the anodic polarization from the

40-1200 PCFC are significantly larger than that of the 50-1300 PCFC because it is more difficult to deliver fuels through smaller pores and to find appropriate sites for charge transfer and transport reactions. Changes in the Ni content in the composition range of 40–50 vol% may have some effect on the polarization resistance, but any change in the values is reported to have only minor impact on the electrode performance[32]. This observation supports that the main cause of the extremely reduced polarization impedance, seen for 50-1300 PCFC in supplementary Fig. 7b, is improvement in the microstructure rather than increase in the Ni content. This finding supports the conclusion that annealing at 1300 °C is necessary to achieve optimal power output in our case.

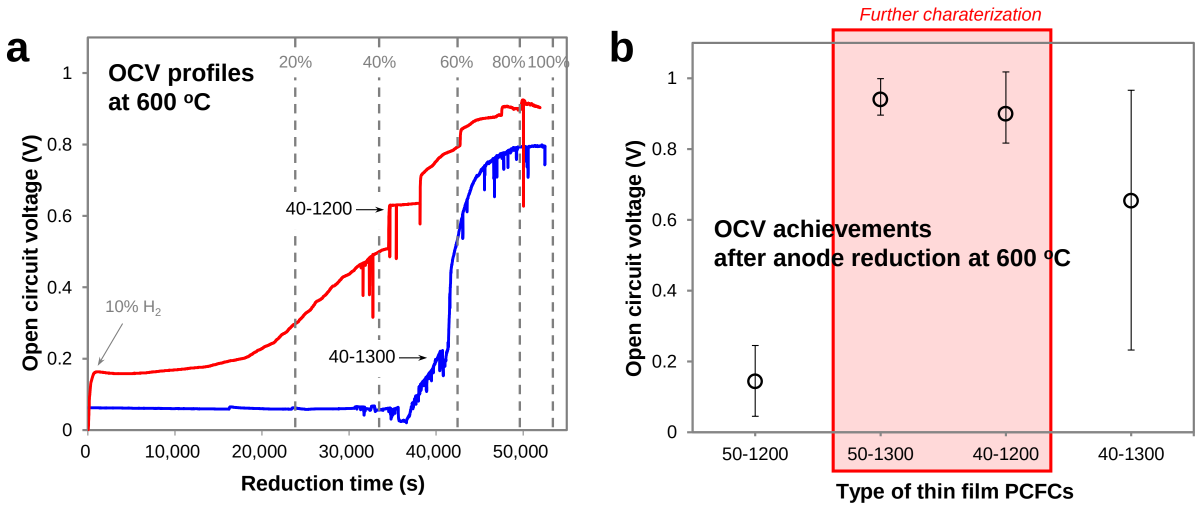

Supplementary Figure 6. OCV comparison of fabricated PCFCs. a, Comparison of OCV profiles obtained from 40-1200 and 40-1300 PCFCs during anode reduction at 600 °C. OCV profiles obtained from 50-1200 and 50-1300 PCFCs are compared in Fig. 3a in the main manuscript. **b**, OCVs after obtained from at least three samples of each PCFC type anode reduction at 600 °C. Error bars presents a gap between the maximum and minimum values.

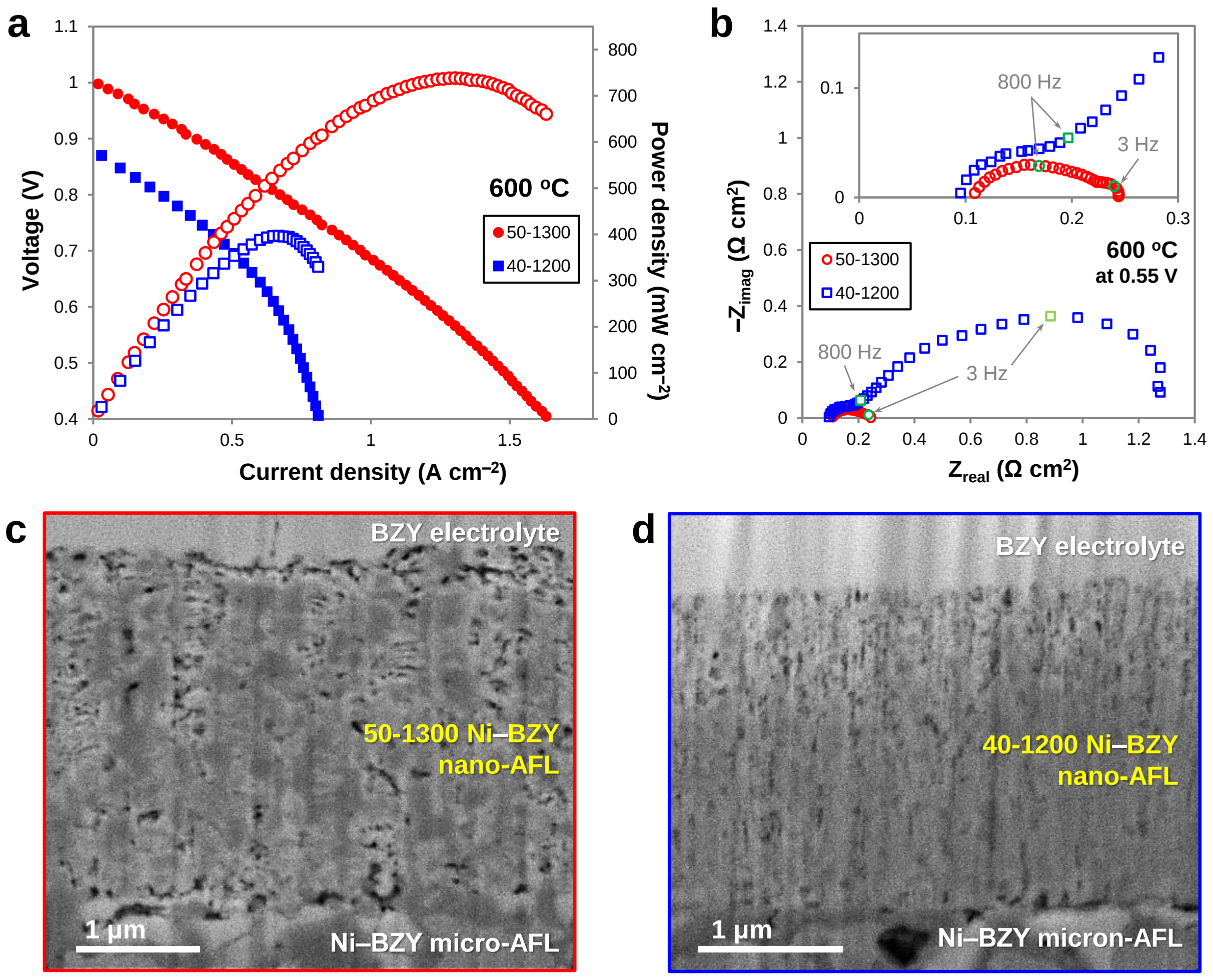

Supplementary Figure 7. Comparison of PCFCs with high and reproducible OCV achievement. **a**, Comparison of power curves obtained from 50-1300 and 40-1200 PCFCs at 600 °C. **b**, Comparison of AC impedance spectra measured at a cell voltage of 550 mV of the data in (**a**). **c**,**d**, Cross sectional FIB-SEM images of Ni of (**a**) 50-1300 and (**b**) 40-1200 PCFCs after the fuel cell test. The pore sizes are estimated as ~110 nm from 50-1300 nano-AFL (**a**) and 10–20 nm from 40-1200 nano-AFL (**b**).

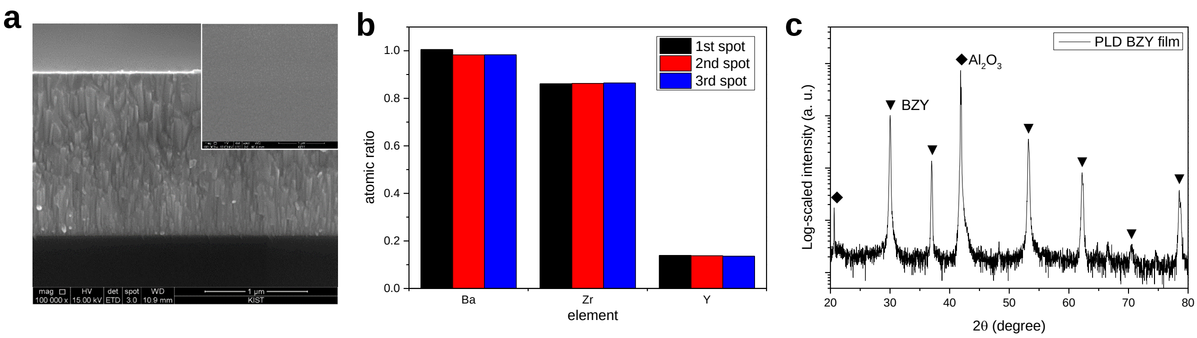

Supplementary Figure 8. Properties of BZY film fabricated on sapphire substrates under the same PLD conditions as those for fabrication of the BZY electrolyte on the tested PCFCs in the main article: a, Cross-sectional and surface (embedded) SEM images. **b**, Compositional data measured at three different positions on the surface of the film by using EDS-equipped SEM. **c**, X-ray diffraction data.

Supplementary Methods

Chemical stability tests. For the powder tests, four different PC powders, $BaCe_{0.8}Y_{0.2}O_{3-\delta}$ (BCY), $BaCe_{0.7}Zr_{0.1}Y_{0.1}Yb_{0.1}O_{3-\delta}$ (BCZYYb), $BaCe_{0.55}Zr_{0.3}Y_{0.15}O_{3-\delta}$ (BCZY), and $BaZr_{0.85}Y_{0.15}O_{3-\delta}$ (BZY) were prepared. The solid-state reaction method was used for the powder synthesis of BCY, BCZYYb, and BZY. The raw materials of $BaCO_3$, ZrO_2, Y_2O_3, and Yb_2O_3 were mixed by using zirconia balls for 24 h, and then calcined at 1300 °C for 10 h. The calcination was fulfilled repeatedly until a single-phase appeared for each powder. For the BCZY powder, a commercialized powder (K-ceracell Tech.) was used. For evaluation of chemical stability against carbon contamination, the prepared powders were exposed to a flowing gas mixture of CO-CO_2 (50 vol% each) with a flow rate of 200 sccm in a quartz-tube heated at 600 °C for 150 h. The phase of the powders was examined before and after the heat treatment using X-ray diffraction analysis.

For the fuel cell test, the BZY and the BCZYYb pallet cells were prepared using the synthesized powders pressed at 200 MPa followed by sintering at 1700 °C and 1500 °C for 10 h, respectively. The sintered pellets were then grinded to a thickness of 750 μm. Porous Pt and Pt–Ru layers were deposited by radio-frequency sputtering as the cathode and anode, respectively. During the cell tests, vaporized methanol with a water content of 30% was supplied by N_2 carrier gas on the anode side, and the cathode side was open to the air. A constant current density of 3.3 mA cm^{-2} was applied at an operating temperature of 450 °C.

Optimization of nano-anode functional layers. Thin-film nano-AFLs with thicknesses of ~3 μm were deposited on the tape-casted anode supports by PLD using NiO–BZY targets with after-reduction Ni contents of 40, 50, and 60 vol%. Then, nano-AFLs were post-annealed in ambient air at various temperatures (1100, 1200, and 1300 °C) for 1 h with a uniform heating and cooling rate of 2 °C min^{-1}. The other elements including the thin BZY electrolyte, the NiO–BZY anode support and the LSC cathode were fabricated following the

conditions mentioned in the main manuscript. The reduction for the microstructural optimization of nano-AFL was conducted in a tube furnace under the flow of 4% H_2−Ar at 650 °C for 10 h.

Characterization. The characterization tools and processes as introduced in the main manuscript were also used for the materials in the supplementary information. In addition, crystallinity and composition data were obtained by using X-ray diffraction (D/MAX-2500, Rigaku) and EDS-equipped SEM, respectively, in the main article. A FIB (Helios NanoLab 600, FEI) was used to examine the cross-sectional microstructure of the tested PCFCs in a milled plane combined with SEM embedded in it.

Supplementary References

1. Shim, J. H., Park, J. S., An, J., Gur, T. M., Kang, S. & Prinz, F. B. Intermediate-temperature ceramic fuel cells with thin film yttrium-doped barium zirconate electrolytes. *Chem. Mater.* **21**, 3290–3296 (2009).

2. Kim, Y. B., Gur, T. M., Kang, S., Jung, H. J., Sinclair, R. & Prinz, F. B. Crater patterned 3-D proton conducting ceramic fuel cell architecture with ultra thin Y:$BaZrO_3$ electrolyte. *Electrochem. Commun.* **13**, 403–406 (2011).

3. Su, P.-C. & Prinz, F. B. Cup-shaped yttria-doped barium zirconate membrane fuel cell array. *Microelectron. Eng.* **88**, 2405–2407 (2011).

4. Bae, K., Jang, D. Y., Jung, H. J., Kim, J. W., Son, J. W. & Shim, J. H. Micro ceramic fuel cells with multilayered yttrium-doped barium cerate and zirconate thin film electrolytes. *J. Power Sources* **248**, 1163–1169 (2014).

5. Kang, S., Heo, P., Lee, Y. H., Ha, J., Chang, I. & Cha, S.-W. Low intermediate temperature ceramic fuel cell with Y-doped $BaZrO_3$ electrolyte and thin film Pd anode on porous substrate. *Electrochem. Commun.* **13**, 374–377 (2011).

6. Park, J., Paek, J. Y., Chang, I., Ji, S., Cha, S. W. & Oh, S. I. Pulsed laser deposition of Y-doped $BaZrO_3$ thin film as electrolyte for low temperature solid oxide fuel cells. *CIRP Ann. – Manuf. Techn.* **62**, 563–566 (2013).

7. Pergolesi, D., Fabbri, E. & Traversa, E. Chemically stable anode-supported solid oxide fuel cells based on Y-doped barium zirconate thin films having improved performance. *Electrochem. Commun.* **12**, 977–980 (2010).

8. Xiao, J., Sun, W., Zhu, Z., Tao, Z. & Liu, W. Fabrication and characterization of anode-supported dense $BaZr_{0.8}Y_{0.2}O_{3-\delta}$ electrolyte membranes by a dip-coating process. *Mater. Lett.* **73**, 198–201 (2012).

9. Sun, W., Yan, L., Shi, Z., Zhu, Z. & Liu, W. Fabrication and performance of a proton-conducting solid oxide fuel cell based on a thin $BaZr_{0.8}Y_{0.2}O_{3-\delta}$ electrolyte membrane. *J. Power Sources* **195**, 4727–4730 (2010).

10. Bi, L., Fabbri, E., Sun, Z. & Traversa, E. Sinteractive anodic powders improve densification and electrochemical properties of $BaZr_{0.8}Y_{0.2}O_{3-\delta}$ electrolyte films for anode-supported solid oxide fuel cells. *Energ. Environ. Sci.* **4**, 1352–1357 (2011).

11. Guo, Y., Lin, Y., Ran, R. & Shao, Z. Zirconium doping effect on the performance of proton-conducting $BaZr_yCe_{0.8-y}Y_{0.2}O_{3-\delta}$ ($0.0 \le y \le 0.8$) for fuel cell applications. *J. Power Sources* **193**, 400–407 (2009).

12. Bi, L., Fabbri, E., Sun, Z. & Traversa, E. A novel ionic diffusion strategy to fabricate high-performance anode-supported solid oxide fuel cells (SOFCs) with proton-conducting Y-doped $BaZrO_3$ films. *Energ. Environ. Sci.* **4**, 409–412 (2011).

13. Bae, H., Choi, J., Kim, K. J., Park, D. & Choi, G. M. Low-temperature fabrication of protonic ceramic fuel cells with $BaZr_{0.8}Y_{0.2}O_{3-\delta}$ electrolytes coated by aerosol deposition method. *Int. J. Hydrogen Energ.* **40**, 2775–2784 (2015).

14. Zhu, Z., Sun, W., Shi, Z. & Liu, W. Proton-conducting solid oxide fuel cells with yttrium-doped barium zirconate electrolyte films sintered at reduced temperatures. *J. Alloy Compd.* **658**, 716–720 (2016).

15. Sun, Z., Fabbri, E., Bi, L. & Traversa, E. Lowering grain boundary resistance of $BaZr_{0.8}Y_{0.2}O_{3-\delta}$ with $LiNO_3$ sintering-aid improves proton conductivity for fuel cell operation. *Phys. Chem. Chem. Phys.* **13**, 7692–7700 (2011).

16. Fabbri, E., Bi, L., Tanaka, H., Pergolesi, D. & Traversa, E. Chemically stable Pr and Y co-doped barium zirconate electrolytes with high proton conductivity for intermediate-temperature solid oxide fuel cells. *Adv. Funct. Mater.* **21**, 158–166 (2011).

17. Sun, Z., Fabbri, E., Bi, L. & Traversa, E. Electrochemical properties and intermediate-temperature fuel cell performance of dense yttrium-doped barium zirconate with calcium addition. *J. Am. Ceram. Soc.* **95**, 627–635 (2012).

18. Fabbri, E., Bi, L., Rupp, J. L. M., Pergolesi, D. & Traversa, E. Electrode tailoring improves the intermediate temperature performance of solid oxide fuel cells based on a Y and Pr co-doped barium zirconate proton conducting electrolyte. *RSC Adv.* **1**, 1183–1186 (2011).

19. Sun, W., Zhu, Z., Shi, Z. & Liu, W. Chemically stable and easily sintered high-temperature proton conductor $BaZr_{0.8}In_{0.2}O_{3-\delta}$ for solid oxide fuel cells. *J. Power Sources* **229**, 95–101 (2013).

20. Bi, L., Fabbri, E., Sun, Z. & Traversa, E. Sinteractivity, proton conductivity and chemical stability of $BaZr_{0.7}In_{0.3}O_{3-\delta}$ for solid oxide fuel cells (SOFCs). *Solid State Ionics* **196**, 59–64 (2011).

21. Liu, Y., Guo, Y., Ran, R. & Shao, Z. A new neodymium-doped $BaZr_{0.8}Y_{0.2}O_{3-\delta}$ as potential electrolyte for proton-conducting solid oxide fuel cells. *J. Membr. Sci.* **415–416**, 391–398 (2012).

22. Choi, S. M., Lee, J.-H., Ji, H. I., Yoon, K. J., Son, J.-W., Kim, B.-K., Je, H. J., Lee, H.-W. & Lee J.-H. Fabrication and characterization of $Ba(Zr_{0.84}Y_{0.15}Cu_{0.01})O_{3-\delta}$ electrolyte-based protonic ceramic fuel cells. *Ceram. Int.* **39**, 9605–9611 (2013).

23. Sun, W., Liu, M. & Liu, W. Chemically stable yttrium and tin co-doped barium zirconate electrolyte for next generation high performance proton-conducting solid oxide fuel cells. *Adv. Energy. Mater.* **3**, 1041–1050 (2013).

24. Sun, W., Shi, Z., Liu, M., Bi, L. & Liu, W. An easily sintered, chemically stable, barium zirconate-based proton conductor for high-performance proton-conducting solid oxide fuel cells. *Adv. Funct. Mater.* **24**, 5695–5702 (2014).

25. Luisetto, I., Licoccia, S., D'Epifanio, A., Sanson, A., Mercadelli, E. & Di Bartolomeo, E. Electrochemical performance of spin coated dense $BaZr_{0.80}Y_{0.16}Zn_{0.04}O_{3-\delta}$ membranes. *J. Power Sources* **220**, 280–285 (2012).

26. Kwon, C. W., Son, J. W., Lee, J. H., Kim, H. M., Lee, H. W. & Kim, K. B. High-performance micro-solid oxide fuel cells fabricated on nanoporous anodic aluminum oxide templates. *Adv. Funct. Mater.* **21**, 1154–1159 (2011).

27. Muecke, U. P., Graf, S., Rhyner, U. & Gauckler, L. J. Microstructure and electrical conductivity of nanocrystalline nickel- and nickel oxide/gadolinia-doped ceria thin films. *Acta Materialia* **56**, 677–687 (2008).

28. Noh, H.-S., Son, J.-W., Lee, H., Ji, H.-I., Lee, J.-H. & Lee, H.-W. Suppression of Ni agglomeration in PLD fabricated Ni-YSZ composite for surface modification of SOFC anode. *J. Eur. Ceram. Soc.* **30**, 3415–3423 (2010).

29. Dasari, H. P., Park, S.-Y., Kim, J., Lee, J.-H., Kim, B.-K., Je, H.-J., Lee, H.-W. & Yoon, K. J. Electrochemical characterization of Ni–yttria stabilized zirconia electrode for hydrogen production in solid oxide electrolysis cells. *J. Power Sources* **240**, 721–728 (2013).

30. Primdahl, S. & Mogensen, M. Oxidation of hydrogen on Ni/yttria-stabilized zirconia cermet anodes. *J. Electrochem. Soc.* **144**, 3409–3419 (1997).

31. Adler, S. B., Lane, J. A. & Steele, B. C. H. Electrode kinetics of porous mixed-conducting oxygen electrodes. *J. Electrochem. Soc.* **143**, 3554–3564 (1996).

32. Bi, L., Fabbri, E., Sun, Z. & Traversa, E. $BaZr_{0.8}Y_{0.2}O_{3-\delta}$-NiO composite anodic powders for proton-conducting SOFCs prepared by a combustion method. *J. Electrochem. Soc.* **158**, B797–B803 (2011).
